# Bone Health in Triathletes: Skeletal Loading, Low Energy Availability, and Bone Stress Injury—A Critical Narrative Review

**DOI:** 10.3390/medicina62101894

**Published:** 2026-09-30

**Authors:** Po-Yuan Lin, Ooi Yean Su, Wen-Tien Wu, Ru-Ping Lee, Ting-Kuo Yao, Kuang-Ting Yeh

**Affiliations:** 1 Department of Orthopedics, Hualien Tzu Chi Hospital, Buddhist Tzu Chi Medical Foundation, Hualien 970473, Taiwan; barny585@tzuchi.com.tw (P.-Y.L.); timwu@tzuchi.com.tw (W.-T.W.); 2 Department of Medical Education, Hualien Tzu Chi Hospital, Buddhist Tzu Chi Medical Foundation, Hualien 970473, Taiwan; u103022514@cmu.edu.tw; 3 Institute of Medical Sciences, Tzu Chi University, Hualien 970374, Taiwan; fish@gms.tcu.edu.tw; 4 School of Medicine, Tzu Chi University, Hualien 970374, Taiwan; 5 Graduate Institute of Clinical Pharmacy, Tzu Chi University, Hualien 970374, Taiwan

**Keywords:** triathlon, bone health, bone stress injury, low energy availability, Relative Energy Deficiency in Sport, bone mineral density, endurance athlete, narrative review

## Abstract

*Background and Objectives:* Triathlon combines low-impact swimming and cycling with repetitive running and high energy demand. We evaluated triathlon-specific evidence on skeletal adaptation, low energy availability (LEA), Relative Energy Deficiency in Sport (REDs), and bone stress injury (BSI). *Materials and Methods:* We purposively selected evidence from PubMed/MEDLINE through 11 August 2026 and citation tracking. The search returned 233 records; 25 primary reports representing approximately 20–22 independent cohorts were synthesized, with the consensus literature providing the context. *Results:* Most studies were small, cross-sectional, and heterogeneous. No uniform bone mineral density deficit emerged; findings varied by site, sex, age, body size, competitive level, race distance, and modality. One four-year cohort of 50 elite short-course athletes found substantial BSI burden and a higher rate in females, but its single-program setting limits generalization. Evidence on female LEA/Triad/REDs relied mainly on questionnaires, dietary assessments, cross-sectional imaging, and case reports; evidence on males and interventions was sparse. Biomarkers, areal bone mineral density, structural imaging, risk screens, and BSI were not interchangeable. *Conclusions:* Evidence does not support universal imaging, biomarker testing, or supplementation; individualized assessment is preferable. Prospective, preregistered, sex- and age-inclusive studies should align discipline-specific exposure, repeated energy assessment, site-specific skeletal measures, and imaging-verified BSI.

## 1. Introduction

Triathlon is a single sport composed of three mechanically dissimilar disciplines. Swimming and cycling permit high aerobic workloads with little ground-reaction impact, whereas running provides repetitive weight-bearing strain and accounts for much lower-extremity overuse injury. In the only multi-season prospective surveillance cohort, bone stress injury (BSI) produced the greatest injury burden; however, the cohort comprised 50 elite short-course athletes from one Australian program, so the finding is not a sport-wide estimate [1]. Skeletal exposure also depends on discipline distribution, race distance, age at specialization, strength-training exposure, recovery, body composition, nutrition, and previous injury. A triathlete is therefore neither simply a runner with additional training nor a cyclist who occasionally runs.

Bone health is similarly multidimensional. Bone stress injury denotes a load-related continuum from early stress reaction to stress fracture. This review uses BSI for the continuum and preserves ‘stress fracture’ when that was the source study’s reported or self-reported outcome. Areal bone mineral density (aBMD) and bone mineral content (BMC) measured by dual-energy X-ray absorptiometry (DXA) describe mineral status but do not capture all aspects of geometry, microarchitecture, tissue quality, or site-specific strength. Bone-turnover markers reflect remodeling over a short interval and are sensitive to sampling time, feeding, exercise, and circadian variation. BSI is a clinical endpoint arising when cumulative loading and recovery exceed local skeletal capacity. These domains overlap biologically but answer different questions; a normal aBMD group mean does not exclude substantial BSI burden, and an acute biomarker change does not prove long-term adaptation.

Energy availability provides an additional explanatory layer. LEA occurs when dietary energy remaining after exercise is insufficient to support normal physiological function. Sustained or problematic LEA may contribute to REDs, with possible skeletal, reproductive, endocrine, metabolic, hematologic, immune, cardiovascular, and psychological consequences in female and male athletes [2]. Nevertheless, energy intake, exercise expenditure, and fat-free mass are difficult to measure precisely in free-living athletes. A calculated value, symptom questionnaire, menstrual disturbance, or isolated hormonal result should therefore be interpreted as part of a clinical pattern rather than as a stand-alone diagnosis.

The direct triathlon literature is dispersed. One review has addressed performance nutrition, training, competition, and recovery broadly [3]; a multi-sport scoping review mapped BMD across numerous sports but excluded studies characterized by LEA, menstrual disorders, or REDs [4]; and a systematic review and meta-analysis examined LEA/REDs across sports without resolving triathlon-specific loading and injury pathways [5]. None was designed to critically integrate structural bone outcomes, BSI surveillance, and energy-deficiency phenotypes specifically in triathletes.

The purpose of this critical narrative review is to determine what can—and cannot—be concluded from triathlon-specific evidence on skeletal adaptation, LEA/REDs, and BSI. We focus on four questions: whether triathletes show a consistent skeletal phenotype; how this phenotype relates to clinical BSI; what the direct evidence shows about sex-specific energy and endocrine risk; and which assessments or preventive actions are currently defensible.

## 2. Approach to Evidence Identification and Critical Synthesis

This article was developed as a critical narrative review. Narrative methodology was chosen because the evidence spans cross-sectional DXA and structural imaging, prospective injury surveillance, acute physiology, questionnaires, nutritional assessment, and individual cases, with outcomes that cannot be validly pooled. We followed the principles underlying the Scale for the Assessment of Narrative Review Articles (SANRA): justification of importance, a focused aim, description of the literature identification, explicit referencing, scientific reasoning, and presentation of relevant endpoint data [6,7,8]. Study-level population, design, outcome, contribution, and main bias/limitation data are reported in Table S1. A completed author self-assessment is provided in Table S2, which maps each SANRA item to the corresponding evidence in the revised manuscript; this reporting checklist is not a substitute for a design-specific risk-of-bias assessment. The intended scope and interpretation of this critical narrative review are summarized in the Supplementary Note.

PubMed/MEDLINE was searched from inception through 11 August 2026. The reproducible strategy combined the Medical Subject Heading for triathlon and triathlon/triathlete free-text terms with controlled vocabulary and free-text terms for bone, aBMD/BMC, geometry and turnover, stress reaction/fracture, BSI, energy availability, REDs, the Female Athlete Triad, menstrual or reproductive function, testosterone, estradiol, calcium, and vitamin D. Arthroplasty, prosthesis, and implant terms were excluded. The development search retrieved 233 records. Backward and forward citation tracking of directly relevant reports and key reviews supplemented the database search; the complete reproducible syntax, date limit, and development-search count are reported in the Supplementary Methods (File S1).

Selection was purposive rather than systematic. All 233 retrieved titles and abstracts were reviewed during manuscript development. Twenty-five peer-reviewed triathlon-specific primary reports were ultimately retained after database screening and citation tracking. Priority was given to human research in which triathletes constituted the entire sample or an extractable subgroup and at least one eligible skeletal, BSI, energy-availability, menstrual, endocrine, or bone-related nutritional outcome was reported. The main exclusion categories were non-triathlon populations, no eligible outcome, implant/arthroplasty uses of ‘Triathlon’, and reviews or opinions used only for context. A prospective full-text exclusion log and source-specific yield from citation tracking were not maintained; therefore, an exact full-text count cannot be reconstructed without false precision. The grey literature, conference abstracts, theses, and unpublished federation data were not systematically searched. The record counts, selection account, eligibility emphasis, main exclusion categories, and the reason an exact full-text count is unavailable are detailed in the Supplementary Methods.

Evidence was synthesized by outcome domain and weighed using a structured narrative appraisal framework: directness to triathlon, design, sample size, selection and recall bias, exposure measurement, endpoint validity, temporal alignment, confounding, and cohort independence. Prospective medically recorded BSI surveillance was considered more direct for clinical burden than retrospective recall. DXA aBMD, geometry, and HR-pQCT were interpreted according to the construct measured; questionnaires were treated as risk screens; and case reports illustrated plausible sequences rather than frequency or causality. No numeric quality score or formal design-specific risk-of-bias tool was applied because the included designs were highly heterogeneous.

The 25 retained primary reports [1,9,10,11,12,13,14,15,16,17,18,19,20,21,22,23,24,25,26,27,28,29,30,31,32] correspond to approximately 20–22 potentially independent cohort groups after probable or possible overlap among the Duncan [11,12], Maïmoun [14,16,17], Nevill [15,31], and Gama [26,32] publications. Because recruitment dates and participant identifiers were incomplete, this is an uncertainty range rather than an exact count. Table 1 maps all retained reports by evidence domain; Table S1 provides the report-level inventory, cohort flags, population and sample characteristics, competitive level/distance, design, primary outcome/site, and principal contribution and limitation.

## 3. Triathlon as a Distinct Skeletal Exposure

Mechanical loading influences bone through the magnitude, rate, direction, distribution, and novelty of strain, not simply through training duration. Swimming offers limited gravitational loading; cycling transfers force through constrained positions and is largely non-impact; running introduces cyclic impact and muscle-generated strain. Combining the three may produce high cardiorespiratory fitness and substantial muscular loading without the same osteogenic stimulus expected from multidirectional impact sports. Multi-sport comparisons have indeed shown that skeletal associations vary by sport and anatomical site [10,15,31].

This mixed exposure can generate apparently contradictory findings. Running may support local adaptation while simultaneously accumulating damage when volume increases faster than remodeling capacity. Cycling and swimming increase total exercise energy expenditure and may displace recovery or resistance training without adding equivalent impact. A favorable tibial or femoral adaptation in one compartment does not guarantee equivalent protection at the sacrum, pelvis, metatarsals, or femoral neck. Accordingly, whole-body BMD is a poor summary of the loading problem faced by a specific bone.

Training history and format also matter. Adolescent participation occurs during peak bone-mass acquisition, whereas masters athletes may carry decades of training, different endocrine exposures, and age-related bone loss. Short-course elite athletes experience high-intensity running and structured programs; long-course and Ironman athletes accumulate markedly greater duration and fueling demands; recreational athletes vary widely in coaching, strength work, and recovery. Available reports rarely stratify simultaneously by race distance, sex, age, competitive level, discipline-specific exposure, strength work, and previous BSI. Age-stratified evidence is too sparse to support a separate age-specific synthesis. The phrase ‘triathlon effect’ therefore conceals substantial biological and behavioral heterogeneity.

**Figure 1:**
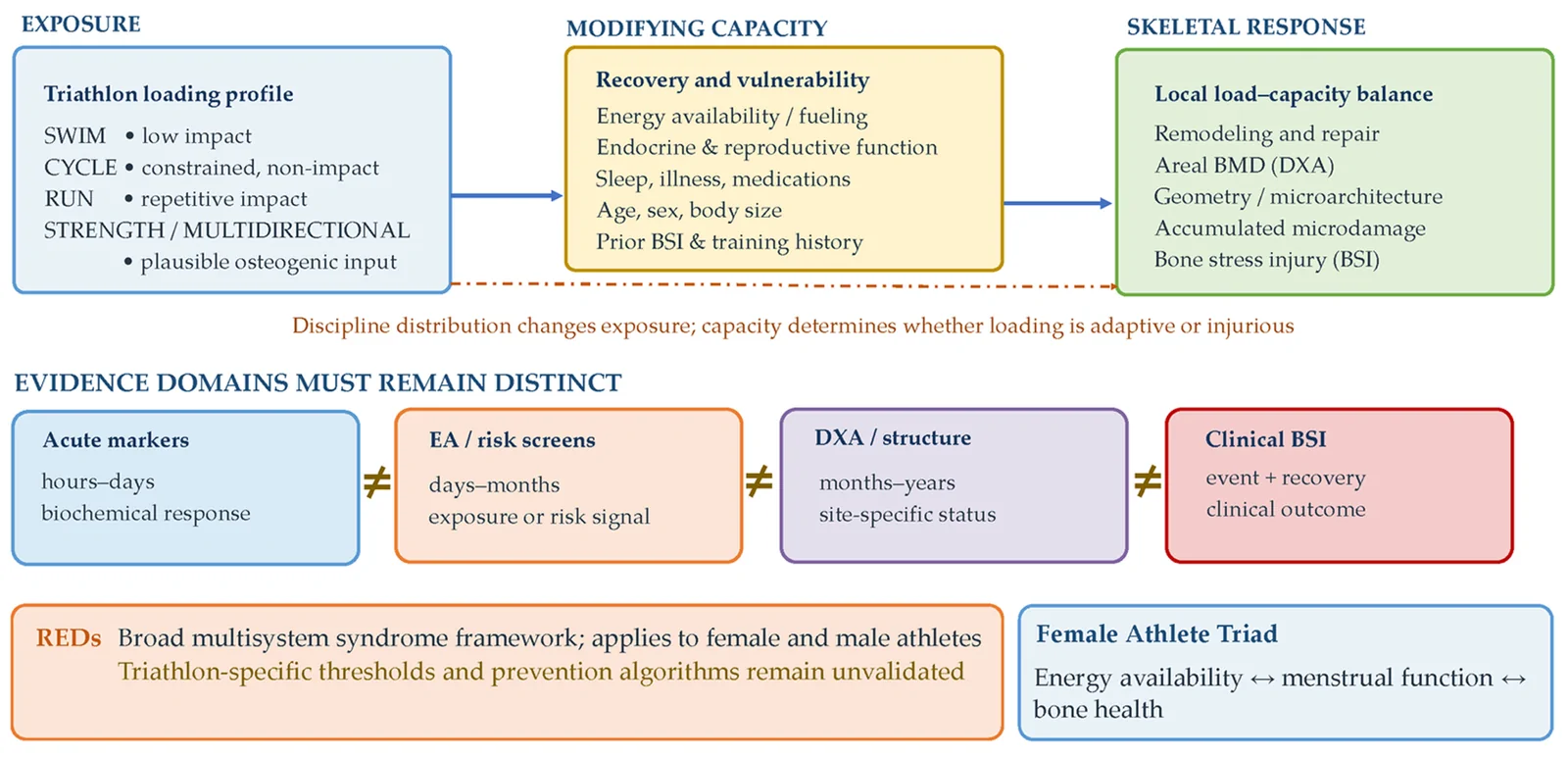
Evidence-bounded conceptual model of bone health in triathlon. Mechanical exposure and recovery capacity jointly influence local skeletal adaptation and injury. Acute biomarkers, energy-availability estimates or risk screens, DXA/structural imaging, and clinical BSI operate over different time scales and should not be substituted for one another. REDs is a broad multisystem framework; the Female Athlete Triad specifically links energy availability, menstrual function, and bone health. Colors distinguish conceptual domains without quantitative or ordinal meaning; lines and arrows denote conceptual relationships, “≠” indicates non-interchangeable evidence domains, and “↔” denotes interrelated Female Athlete Triad components. None of these elements implies proven tri-athlon-specific causality.

Energy availability interacts with this mechanical environment. A loading stimulus requires resources for remodeling and recovery; chronic under-fueling may blunt beneficial adaptation or prolong repair even when running provides an osteogenic signal. Conversely, a triathlete with adequate availability and progressive loading should not automatically be classified as having impaired bone health merely because two disciplines are non-weight-bearing. The most defensible model is conditional: skeletal outcome reflects local loading history multiplied by recovery capacity, with LEA, endocrine state, sleep, illness, medications, and previous injury modifying that capacity. The proposed relationships are summarized in Figure 1.

## 4. Bone Mass, Geometry, and Turnover

Early cross-sectional studies do not identify a uniform triathlon BMD deficit. Male triathletes in Smith and Rutherford’s small comparison had spine and total-body BMD broadly comparable with controls despite lower testosterone [9]. In adolescent females, Duncan and colleagues found sport- and site-dependent BMD and mid-femur geometry rather than a simple whole-body triathlon advantage or disadvantage [11,12]. Multi-sport analyses similarly demonstrate that conclusions depend on anatomical site, comparator sport, and adjustment for body size [10,15,31]. These results argue against labeling triathlon globally as ‘osteopenic’ or ‘osteogenic’. Report-level population, design, outcome, and limitation details for the studies discussed in this section are provided in Table S1.

Body-size adjustment is particularly consequential. DXA provides a two-dimensional areal estimate and is influenced by bone size; smaller athletes may appear to have lower values even when volumetric structure is not proportionally deficient. The 2024 comparison of female amateur triathletes and inactive controls showed that interpretation changed when body size was considered [29]. Allometric research models may improve group comparison [31], but they are not automatically clinical diagnostic standards. In practice, age- and sex-appropriate reference data, longitudinal change, least significant change, fracture history, menstrual/endocrine context, and site-specific symptoms remain essential.

Geometry and microarchitecture add information that BMD alone cannot supply. Mid-femur geometry in adolescent athletes varied across loading modalities [12], and tibial geometry was associated with stress reactions in elite-development triathletes [20]. HR-pQCT work in female long-distance triathletes suggested that exercise-associated structural advantages may be attenuated in the presence of low EA [26]. These findings are biologically coherent but remain cross-sectional and derived from small samples. They identify mechanisms and candidate markers, not validated triathlon-specific screening cut-points.

### 4.1. Seasonal and Training-Related Change

Longitudinal evidence is reassuring only within narrow boundaries. Across 24 weeks, McClanahan and colleagues observed no adverse total-body or leg aBMD change in 21 competitive triathletes, with an arm-aBMD increase in men [13]. In seven young male triathletes followed for 32 weeks, lumbar-spine aBMD increased by 1.9% (*p* < 0.05), while bone mineral status was not uniformly impaired [17]. A simulated-altitude training program in 18 trained men did not demonstrate a clear short-term aBMD benefit [21]. These studies show that measurable bone loss is not inevitable during a season, but their duration, sample size, and limited BSI ascertainment cannot exclude cumulative or site-specific risk.

Areal BMD changes slowly, and short observation windows may be insensitive to clinically relevant remodeling or microdamage. Training-season studies are also vulnerable to regression to the mean, measurement precision, unmeasured dietary change, and selective retention of healthier athletes. Absence of a group-level decline should therefore be stated as exactly that—not proof that every participant maintained adequate skeletal adaptation or that BSI risk was low.

### 4.2. Acute Bone-Turnover Responses

Bone-turnover markers offer temporal sensitivity but are easy to overinterpret. Following a 226-km Ironman race, nine amateur men showed transient changes in osteocalcin without a corresponding rise in C-terminal telopeptide [25]. The result describes a short post-race biochemical response under a specific sampling protocol. It does not demonstrate net bone formation, improved bone strength, or protection from future BSI. Marker concentrations may reflect hemoconcentration, feeding, circadian rhythm, assay variation, and the timing of exercise and recovery.

The seasonal study by Maïmoun and colleagues reported a decrease in a formation marker despite a 1.9% lumbar-spine aBMD increase [17]. This discordance is not necessarily paradoxical: biomarkers and DXA integrate biology over different time scales and at different levels. A rigorous review must resist converting every direction of change into a clinical benefit or harm narrative.

## 5. Bone Stress Injury: The Clinically Decisive Outcome

The most clinically direct evidence comes from one prospective surveillance study of 50 elite Australian short-course triathletes followed across four seasons. Twenty athletes (40.0%) sustained at least one BSI. BSI burden was 31.38 time-loss days per 365 athlete-days (95% CI 24.42–38.34), and the female-to-male BSI incidence-rate ratio was 2.99 (95% CI 1.26–7.07; *p* = 0.013) [1]. These are precise study-specific estimates, not prevalence estimates for recreational, long-course, junior, or masters triathletes. Nutritional exposure was not measured contemporaneously, and the report’s sex contrast should not be converted into unreported absolute sex-specific rates. The corresponding design and setting, outcome definition, contribution, and main limitation are summarized in Table S1.

Retrospective evidence is directionally consistent but methodologically weaker. Among 86 German elite triathletes, 20 reported at least one stress fracture, with associations involving sex and menstrual or nutritional factors [24]. Recall, survivor selection, variable imaging confirmation, and uncertain timing between risk factors and injury prevent causal estimates. Self-reported lifetime stress fracture should not be pooled conceptually with prospectively verified BSI incidence or time-loss burden.

Local skeletal structure may modify injury susceptibility. The tibial geometry study linked regional cortical features with stress reactions in elite-development triathletes [20], while cross-sectional HR-pQCT work connected low EA with attenuated bone characteristics and included fracture-history information [26]. These studies support a multifactorial model but cannot determine whether structural findings preceded injury, resulted from altered loading after injury, or reflected a third factor such as body size or training history.

The clinical lesson is that population-average aBMD and BSI burden can diverge. BSI represents a local failure of load–capacity balance and may occur in an athlete whose routine DXA values are not low. Conversely, a low Z-score indicates skeletal concern but does not specify injury site or timing. Risk assessment must integrate symptoms, site, imaging, prior BSI, training change, recovery, energy availability, endocrine and reproductive status, and other causes of impaired bone health, rather than elevating one metric to a universal proxy.

## 6. Low Energy Availability, REDs, and Endocrine Phenotypes

The direct triathlon evidence linking LEA to bone outcomes is plausible but limited. LEA can reduce substrates for recovery and alter reproductive and metabolic signaling, and the IOC REDs framework recognizes skeletal health as an important outcome [2]. Across sports, recent synthesis supports adverse associations between LEA/REDs and multiple health or performance outcomes [5]. Nevertheless, applying cross-sport evidence to triathlon requires explicit acknowledgment that direct triathlon cohorts are small, measurement windows are short, and exposure and outcome are frequently assessed at the same time. All direct triathlon reports supporting this section, together with their population, design, outcome, and main bias/limitation, are catalogued in Table S1.

### 6.1. Evidence in Female Triathletes

Female athletes dominate the direct literature. In a club team of 15 women, dietary deficit and menstrual-history signals were present even though group mean hip and spine aBMD were not uniformly low [18]. The study warns against relying on a mean aBMD value to dismiss risk, but it cannot establish the prevalence of clinically defined LEA or predict injury. In an online survey of 311 adult female triathletes, 24% screened positive for at least one Female Athlete Triad arm [23]. Self-selection, incomplete questionnaire response, and self-report limit diagnostic specificity.

The LEAF-Q has also been applied to 30 female triathletes and identified individuals with LEA-related or menstrual-risk signals [27]. The instrument can organize case finding, but a positive score is not synonymous with measured LEA, REDs, or low bone density. Gastrointestinal symptoms, contraceptive use, menstrual classification, prior illness, and training context can influence responses. Its proper clinical role is to trigger focused assessment rather than close the diagnostic process.

Objective skeletal imaging has strengthened the mechanistic case without resolving causality. Gama and colleagues compared female long-distance triathletes with controls using estimated EA, DXA, and HR-pQCT; potentially favorable exercise-associated bone features appeared attenuated in athletes with low EA [26]. Because exposure was estimated over a limited period and the design was cross-sectional, reverse causation and residual confounding remain possible. The study nevertheless demonstrates why compartment- and site-specific imaging may reveal patterns obscured by whole-body DXA.

Individual cases show severe but non-generalizable phenotypes. An elite junior triathlete with very low estimated EA had endocrine suppression, menstrual dysfunction, and a low lumbar Z-score [22]. A later professional-athlete report described menstrual and reproductive recovery accompanying reduced training and increased intake during the pandemic period [32]. These temporal narratives are clinically coherent and useful for hypothesis generation; they cannot determine population frequency, isolate which intervention component produced recovery, or quantify BSI reduction.

Nutritional assessment of 20 high-performing female amateur triathletes identified dietary patterns relevant to REDs risk but included no objective bone endpoint [30]. Such studies can reveal actionable intake gaps, yet a few recorded days may not represent a chronic training cycle, and reporting accuracy is difficult to verify. Nutrient insufficiency, LEA, and REDs overlap but are not interchangeable: an athlete may have inadequate calcium or vitamin D without LEA, or LEA without a single nutrient deficiency.

### 6.2. Evidence in Male Triathletes

Male-specific evidence is markedly thinner. Small male BMD studies reported lower testosterone without a corresponding group-level BMD deficit [9,14], while a training-camp case series of five elite Japanese men related estimated EA to nocturnal interstitial glucose but included no skeletal outcome [28]. These studies show that male physiology can be measured in the field; they do not identify an LEA threshold that predicts BSI or establish whether transient endocrine changes are adaptive, pathological, or unrelated to bone.

Under-recognition is possible because male athletes lack an obvious menstrual marker and may normalize high training volume, weight cycling, low libido, fatigue, or performance decline. Clinically, delayed recognition matters when persistent endocrine or reproductive symptoms coexist with focal bone pain, recurrent BSI, or impaired recovery, because attributing the pattern to ‘normal training’ may prolong exposure. Conversely, indiscriminate endocrine testing may capture transient physiological variation and promote overdiagnosis. The current triathlon literature cannot quantify male REDs prevalence, define a triathlon-specific endocrine profile, or support universal testosterone testing. Evaluation should therefore be symptom- and history-led, with repeat or laboratory assessment guided by differential diagnosis rather than sport participation alone [2,33].

### 6.3. Measurement and Conceptual Limits

Energy availability is often expressed as dietary energy intake minus exercise energy expenditure, normalized to fat-free mass. Each component contains errors. Self-reported intake is commonly under-recorded, exercise expenditure is model-dependent, and fat-free mass can change. A few measured days may not represent chronic adaptation. Conceptual thresholds should not be treated as perfectly measured universal boundaries [34,35].

Repeated measurements across representative heavy, recovery, and competition blocks, corroborated by physiological and clinical information, are more informative than a single calculated value. REDs is a syndrome framework, not a laboratory result. This review uses ‘REDs’ in accordance with the current IOC nomenclature; ‘RED-S’ is retained only when it appears in a source title or earlier terminology. The IOC REDs Clinical Assessment Tool version 2 uses a stepwise process integrating indicators, severity, differential diagnosis, and sport-participation decisions [33]. The Female Athlete Triad framework remains specifically relevant to energy availability, menstrual function, and bone health, with updated guidance emphasizing individualized screening, diagnosis, treatment, and return-to-play decisions [36,37]. These frameworks guide clinical reasoning but do not create a validated triathlon-specific algorithm.

The distinction between risk phenotype and clinical outcome is fundamental. A short food record, LEAF-Q score, menstrual disturbance, low testosterone, low BMD, and imaging-confirmed BSI can coexist, but none necessarily proves the others. Table 2 states the interpretive boundary for each evidence type and is intended to prevent domain substitution—the recurrent error in which an indirect marker is presented as though it were a clinical endpoint.

## 7. Integrated Clinical Interpretation

Current evidence is insufficient to support universal DXA screening, serial bone-turnover panels, routine hormonal testing, or blanket calcium and vitamin D supplementation for all triathletes. It supports a low threshold for focused assessment when focal bone pain, recurrent BSI, a high-risk injury site, menstrual disturbance, substantive weight loss, restrictive eating, delayed healing, fatigue, endocrine symptoms, or disproportionate performance decline is present. This distinction protects athletes from both under-investigation and indiscriminate testing.

The following recommendations are not evidence-graded. They restate relevant IOC REDs CAT2 and Female Athlete Triad guidance where applicable and combine it with broader BSI management principles; the review’s added contribution is to map those principles to the limits of direct triathlon evidence. Table 3 makes that basis explicit.

For suspected BSI, immediate priorities are anatomical localization, site-appropriate imaging, and protection from loads that threaten progression. Etiologic evaluation should then examine the trajectory of running and total training load, recent race preparation, prior BSI, recovery, energy intake relative to exercise, menstrual or reproductive history, medications, endocrine symptoms, and other medical contributors. A practical metabolic workup should be individualized to injury severity, recurrence, clinical history, and examination rather than applied as a fixed panel [38].

Nutrition management should target adequate total energy and carbohydrate availability around demanding sessions, sufficient protein distribution, and correction of demonstrated micronutrient insufficiency. Calcium and vitamin D are biologically important, but supplementation should reflect dietary intake, laboratory or clinical indication, and safety rather than the assumption that every BSI is a deficiency disorder. When disordered eating or an eating disorder is suspected, treatment requires appropriately trained mental-health and nutrition professionals in addition to sports medicine care.

Loading remains treatment as well as exposure. Eliminating all impact indefinitely may reduce the stimulus needed for skeletal adaptation, whereas premature return can reactivate injury. Rehabilitation therefore aims to rebuild site-appropriate capacity through injury-guided progression, restoration of energy availability, and correction of biomechanical or training errors. Resistance, plyometric, and multidirectional loading are biologically plausible strategies extrapolated from the broader bone-loading literature, but no triathlon-specific trials have shown that they prevent BSI. High-risk sites require more conservative specialist pathways than low-risk sites. The principle that return should not be based on pain alone is likewise extrapolated from general BSI management guidance [38].

## 8. Priorities for Research

The first priority is a prospective, sex-inclusive, multicenter cohort spanning junior, elite short-course, long-course, recreational, and masters triathletes. Sample-size planning should be based on prespecified primary outcomes, and protocols and analysis plans should be preregistered. BSI should be defined by symptoms, anatomical site, imaging confirmation, grade, recurrence, time loss, and exposure-adjusted incidence. Training should be recorded by discipline, intensity, surface, footwear, strength work, internal load, and rapid change rather than reduced to weekly hours.

Second, exposure measurement must improve. Energy intake and exercise expenditure should be assessed repeatedly across representative heavy, recovery, and competition phases, with transparent uncertainty. Menstrual status, hormonal contraception, reproductive and endocrine symptoms, body composition, sleep, illness, and relevant laboratory measures should be temporally aligned with skeletal and injury outcomes. Male studies require sufficient sample size and prespecified endocrine endpoints rather than opportunistic subgroup reporting.

Third, skeletal assessment should match the hypothesis. DXA is useful for clinically interpreting aBMD and longitudinal change; geometry and HR-pQCT can examine site- and compartment-specific adaptation; and magnetic resonance imaging or other appropriate imaging should verify BSI. Bone-turnover markers may clarify mechanisms when sampling is standardized but should not serve as the only endpoint of a prevention trial. Harmonized outcome definitions, acquisition protocols, and reporting would improve future pooling.

Fourth, intervention studies are needed. No controlled triathlon-specific study has established that correcting LEA, altering discipline distribution, adding resistance, plyometric, or multidirectional impact training, or providing a supplement reduces BSI or improves a clinically meaningful bone outcome. These remain plausible strategies, not proven triathlon interventions. Pragmatic trials could test individualized fueling plus strength/loading programs in athletes with prespecified risk phenotypes while monitoring adherence, adverse events, performance, and recurrence.

Finally, reporting should make cohort linkage visible. Several small publications may originate from overlapping samples, and treating each report as independent exaggerates the evidence base. Unique cohort identifiers, recruitment dates, protocol registration, shared core outcomes, sex-disaggregated data, and accessible analytic code would substantially improve synthesis and future meta-analysis. These priority questions and minimum reporting safeguards are summarized in Table 4.

## 9. Strengths and Limitations of This Review

This review’s principal strength is conceptual separation. It integrates the sport-specific literature while preserving distinctions among acute biomarkers, aBMD/BMC, geometry and microarchitecture, BSI, dietary exposure, endocrine findings, and risk screens. Direct triathlon evidence is prioritized, with the broader consensus and endurance literature framing the mechanisms and clinical reasoning. Explicit search and selection reporting (Supplementary Methods), precise endpoint data, the full report-level evidence inventory (Table S1), the conceptual figure, the completed SANRA self-assessment (Table S2), and the explicit interpretation of review scope (Supplementary Note) improve transparency beyond an unstructured overview.

The review is not systematic. PubMed/MEDLINE was the only bibliographic database, selected for the clinically oriented narrative scope; SPORTDiscus, Scopus, Web of Science, and Embase were not searched. Citation tracking partly mitigates but cannot eliminate the risk of missing sports-science reports. The grey literature, conference abstracts, theses, and unpublished federation data were not systematically examined. Screening was not duplicated, a prospective full-text exclusion log was not retained, and no formal design-specific risk-of-bias tool was applied. Narrative weighting therefore remains vulnerable to missed studies and author judgment. Database and selection details are reported in the Supplementary Methods, and the implications of these boundaries for review completeness and permissible claims are stated in the Supplementary Note.

The underlying evidence is also limited. Twenty-five primary reports represent only approximately 20–22 potentially independent cohort groups because several publications may share participants; exact independence could not be verified from incomplete recruitment identifiers. The probable or possible overlap clusters and the report-level design limitations are detailed in Table S1. Most studies are small, single-center, and cross-sectional; LEA/Triad/REDs evidence is concentrated in female cohorts. Definitions, comparators, dietary windows, and BSI ascertainment vary. The 33-year span also crosses substantial changes in DXA hardware/software, reference norms, and availability of HR-pQCT, limiting direct comparability. Publication bias is plausible because small null studies may remain unpublished or receive less emphasis. These constraints preclude pooled LEA/REDs prevalence, causal estimates, validated screening thresholds, or one preventive intervention for all triathletes.

## 10. Conclusions

Triathlon creates a distinctive interaction among high total training demand, limited impact in swimming and cycling, repetitive running strain, and potentially constrained recovery. Direct studies do not show a universal aBMD deficit; findings depend on site, sex, age, body size, competitive level, race distance, comparator, and measurement method. One 50-athlete elite short-course program demonstrated clinically important BSI burden, particularly among women, but this population-specific result should not be generalized to all triathletes. The evidence remains predominantly small, single-center, and cross-sectional, while LEA/Triad/REDs evidence is concentrated in female cohorts. Current evidence favors individualized assessment and multidisciplinary correction of demonstrable risk factors, while prospective preregistered cohorts and controlled prevention trials are needed to establish triathlon-specific causality and benefit.

## Figures and Tables

**Table 1:** Condensed map of all retained triathlon-specific primary reports, grouped by evidence domain.

Evidence Domain	Direct Reports	Populations Represented	Principal Signal	Dominant Limitations
Areal BMD/BMC	[9,10,11,12,13,14,15,16,17,18,19,21,26,29,31] ^†,‡,§,¶^	Adolescent and adult; female and male; elite, competitive, and amateur; mixed distances	No uniform triathlon-wide BMD deficit; findings depend on site, comparator, body size, and training context.	Mostly small cross-sectional samples; inconsistent sites and adjustment; historical DXA differences; potential cohort overlap.
Geometry/microarchitecture	[12,20,26] ^†,¶^	Adolescent/elite-development and adult female long-distance cohorts	Structural measures add information beyond areal BMD and support site-specific interpretation.	Cross-sectional or observational designs; no validated triathlon-specific thresholds.
Seasonal/training change	[13,17,21] ^‡^	Competitive or trained adults; predominantly men	Short follow-up did not show uniform loss; one 32-week study reported a 1.9% lumbar-spine BMD increase.	Small samples, short windows, limited injury ascertainment, and possible measurement error.
Acute bone turnover	[17,25] ^‡^	Young competitive men and amateur male Ironman athletes	Markers can change after a season or race without establishing structural benefit or harm.	Timing, feeding, hemoconcentration, assay variability, and surrogate endpoints.
Clinical BSI/stress fracture	[1,20,24,26] ^¶^	Elite short-course, elite-development, and female long-distance cohorts	BSI is clinically important, but the most precise prospective estimate comes from one 50-athlete elite program.	Population-specific; varying definitions; retrospective recall and incomplete imaging in some reports.
Female LEA/Triad/REDs	[18,22,23,26,27,30,32] ^¶^	Club, junior elite, amateur, long-distance, and professional women	Risk signals and coherent severe phenotypes occur; objective bone outcomes are not consistently measured.	Questionnaires, short dietary windows, cross-sectional designs, and case reports predominate.
Male EA/endocrine evidence	[9,14,16,17,28] ^‡^	Small competitive/elite male samples	Lower testosterone or short-window EA measures do not establish REDs or BSI risk.	Very small samples, sparse skeletal endpoints, and no validated male triathlon threshold.

A report may contribute to more than one domain. ^†^ Potential Duncan cohort overlap [11,12]; ^‡^ potential Maïmoun cohort overlap [14,16,17]; ^§^ potential Nevill dataset overlap [15,31]; ^¶^ possible Gama cohort linkage [26,32]. The 25 reports represent approximately 20–22 potentially independent cohort groups. Full report-level details, including population, competition level/distance, design, outcome, cohort flag, and principal contribution and limitation, are provided in Table S1. Abbreviations: BMC, bone mineral content; BSI, bone stress injury; DXA, dual-energy X-ray absorptiometry; EA, energy availability; LEA, low energy availability; REDs, Relative Energy Deficiency in Sport.

**Table 2:** Evidence domains, typical time horizons, and interpretive boundaries.

Evidence Type	Typical Time Horizon	Can Support	Cannot Establish Alone	Preferred Interpretation
Acute bone-turnover markers	Hours to days	Short-term remodeling response under a defined sampling protocol	Chronic BMD change, bone strength, or lower BSI risk	Report timing, feeding, assay, hydration, and recovery; avoid benefit/harm labels from one value.
DXA BMD/BMC	Months to years	Areal mineral status at a measured site; standardized longitudinal change	Complete strength, microarchitecture, or imminent BSI	Report site, reference data, body size, scanner/software, least significant change, and context.
Geometry or HR-pQCT	Months to years	Distribution, structure, and compartment-specific adaptation	Validated triathlon-specific diagnostic thresholds	Use as complementary structural evidence, not a stand-alone clearance test.
BSI surveillance/history	Event-specific; recovery over weeks to months	Occurrence, site, recurrence, incidence, and burden when definitions are explicit	Etiology attributable to LEA without aligned exposure data	Separate prospective/imaging-verified BSI from recalled stress fracture and nonspecific pain.
LEAF-Q or symptom screen	Instrument-specific recall over weeks to months	Risk signal prompting focused history and assessment	Diagnosis of LEA, REDs, menstrual etiology, or low BMD	Account for contraception, illness, gastrointestinal symptoms, and sport context.
Estimated energy availability	Usually days; chronic inference requires repetition	Time-bounded estimate from intake, exercise expenditure, and fat-free mass	A universally precise chronic exposure or diagnostic cut-point	Repeat across representative training phases and corroborate physiologically and clinically.

Time horizons are approximate and protocol-dependent. Abbreviations: BMC, bone mineral content; BSI, bone stress injury; DXA, dual-energy X-ray absorptiometry; LEA, low energy availability; HR-pQCT, high-resolution peripheral quantitative computed tomography; LEAF-Q, Low Energy Availability in Females Questionnaire; REDs, Relative Energy Deficiency in Sport.

**Table 3:** Evidence-informed, non-validated clinical assessment framework for triathletes.

Clinical Trigger	Focused Assessment	Interpretive Principle	Action Direction	Evidence Basis
Current focal bone pain or suspected BSI	History, examination, site-appropriate imaging, load change, prior BSI, and recovery	Confirm injury and grade/site risk before assigning etiology	Protect the site; coordinate staged return; investigate contributors	Direct triathlon surveillance [1] plus broader BSI clinical guidance [38]
Recurrent BSI, delayed healing, or high-risk site	Energy/nutrition, menstrual/reproductive history, medication, calcium/vitamin D intake, targeted laboratory evaluation, and DXA when indicated	Multiple interacting causes are common; normal group-average BMD does not exclude individual vulnerability	Use multidisciplinary sports medicine, nutrition, endocrine, and bone-health input	Triathlon observational/case evidence [18,22,23,24,26,32] plus consensus/guidance [2,36,37,38]
Menstrual disturbance or endocrine symptoms	Pregnancy status when relevant, menstrual chronology, contraception, endocrine differential, and training/diet change	Do not presume all menstrual or endocrine disturbance is exercise-related	Treat the underlying diagnosis and restore adequate availability/recovery	Direct female triathlon evidence [18,22,23,26,27,32] plus Triad/REDs guidance [2,33,36,37]
Weight loss, dietary restriction, fatigue, impaired recovery, or performance decline	Dietitian-led intake assessment, eating-disorder risk, sleep, illness, discipline distribution, and REDs-informed evaluation	A questionnaire or short food record estimates risk, not diagnosis	Individualize fueling and training; avoid blanket supplementation	Triathlon screening/diet studies [18,23,27,30] plus REDs/Triad guidance [2,33,36,37]
Asymptomatic athlete without risk indicators	Routine history, education, progressive loading, adequate fueling, strength exposure, and recovery surveillance	Evidence is insufficient to support universal testing or supplementation	Escalate when symptoms, history, or risk profile changes	Consensus extrapolation and absence of triathlon intervention trials; not a validated algorithm

This framework is not evidence-graded and has not been validated in triathletes. It combines direct triathlon observations with IOC REDs CAT2, Female Athlete Triad guidance, and broader BSI clinical principles; the evidence-basis column identifies the source of each element.

**Table 4:** Priority research questions and minimum reporting safeguards.

Priority Question	Minimum Design and Reporting Safeguards	Core Measures	Why It Matters
Who develops BSI, and when?	Prospective multicenter cohort; a priori sample-size planning; protocol preregistration; standardized BSI definition	Imaging-verified site/grade, recurrence, time loss, prior injury, and discipline-specific load	Provides generalizable incidence and burden estimates for targeted prevention.
Does LEA precede skeletal deterioration or BSI?	Repeated-measures cohort with temporally aligned exposure and outcomes; prespecified primary endpoints	Repeated EA estimates, diet quality, endocrine/reproductive indicators, DXA/structure, and BSI	Separates cause, consequence, and short-window measurement noise.
Which male phenotype is clinically meaningful?	Adequately powered male cohort with sex-specific hypotheses and harmonized outcomes	Symptoms, libido/reproductive history, endocrine markers, bone outcomes, and BSI	Prevents both under-recognition and inappropriate female-to-male extrapolation.
Can a modifiable program prevent recurrence?	Randomized or strong pragmatic controlled trial; registered protocol; adherence/adverse-event reporting	Individualized fueling, resistance/loading progression, performance, and imaging-verified recurrence	Tests whether plausible strategies produce clinically meaningful triathlon-specific benefit.

Abbreviations: BSI, bone stress injury; DXA, dual-energy X-ray absorptiometry; EA, energy availability.

## Data Availability

No new data were created or analyzed in this study. Data sharing is not applicable to this article.

## Supplementary Materials

The following supporting information can be downloaded at https://www.mdpi.com/article/10.3390/medicina62101894/s1. Supplementary File S1: Supplementary Methods: Literature Identification and Selection. Table S1: Full study-level evidence inventory. Table S2: SANRA author self-assessment. Supplementary Note: Interpretation of review scope.

## Abbreviations

The following abbreviations are used in this manuscript:

aBMD	Areal bone mineral density
BMC	Bone mineral content
BMD	Bone mineral density
BSI	Bone stress injury
DXA	Dual-energy X-ray absorptiometry
EA	Energy availability
HR-pQCT	High-resolution peripheral quantitative computed tomography
LEA	Low energy availability
LEAF-Q	Low Energy Availability in Females Questionnaire
REDs	Relative Energy Deficiency in Sport
RED-S	Earlier/source-title variant retained only when quoting source nomenclature
SANRA	Scale for the Assessment of Narrative Review Articles
