# Supplementary material for: Bone Health in Triathletes: Skeletal Loading, Low Energy Availability, and Bone Stress Injury—A Critical Narrative Review
Source: Medicina (Kaunas). 2026 Sep 30;62(10):1894. doi: 10.3390/medicina62101894 (PMC13706682; doi:10.3390/medicina62101894)
Supplement: Supplementary file 1 [file medicina-62-01894-s001.zip › medicina-4594467-supplementary.pdf]

Supplementary File S1

Bone Health in Triathletes: Skeletal Loading, Low Energy Availability, and Bone Stress Injury—A Critical Narrative Review

Supplementary Methods: Literature Identification and Selection

Database and date: PubMed/MEDLINE was searched from database inception through 11 August 2026. The development search returned 233 records. PubMed records may be re-indexed over time; the stated count refers to the development run with the date limit below.

Exact PubMed syntax:

("Triathlon"[Mesh] OR triathlon*[Title/Abstract] OR triathlete*[Title/Abstract]) AND (bone*[Title/Abstract] OR skeletal[Title/Abstract] OR fracture*[Title/Abstract] OR BMD[Title/Abstract] OR BMC[Title/Abstract] OR "stress reaction"[Title/Abstract] OR "stress reactions"[Title/Abstract] OR "energy availability"[Title/Abstract] OR "energy deficiency"[Title/Abstract] OR "low energy"[Title/Abstract] OR RED-S[Title/Abstract] OR REDs[Title/Abstract] OR "female athlete triad"[Title/Abstract] OR menstrual[Title/Abstract] OR amenorrhea[Title/Abstract] OR testosterone[Title/Abstract] OR estradiol[Title/Abstract] OR calcium[Title/Abstract] OR "vitamin D"[Title/Abstract] OR "reproductive function"[Title/Abstract] OR "Bone and Bones"[Mesh] OR "Bone Density"[Mesh] OR "Fractures, Stress"[Mesh] OR "Female Athlete Triad Syndrome"[Mesh]) NOT (arthroplast*[Title/Abstract] OR prosthe*[Title/Abstract] OR implant*[Title/Abstract]) AND ("1900/01/01"[Date - Publication] : "2026/08/11"[Date - Publication]).

Transparent selection account: Selection was purposive within a critical narrative-review framework, not systematic or PRISMA compliant.

Selection item	Count / status
Records returned by the development PubMed search	233
Titles/abstracts reviewed during manuscript development	233
Primary triathlon-specific reports retained after database review and citation tracking	25
Estimated potentially independent cohort groups after overlap is considered	Approximately 20–22
Exact number of full texts assessed	Not prospectively logged; not reconstructed to avoid false precision

Eligibility emphasis: Peer-reviewed human research was prioritized when triathletes were the entire sample or an extractable subgroup and an eligible skeletal, BSI, energy-availability, menstrual, endocrine, or bone-related nutritional outcome was reported. Mixed-sport evidence was retained only when the triathlon subgroup was interpretable.

Main exclusion categories: Non-triathlon populations; no eligible skeletal, BSI, energy, endocrine, reproductive, or bone-related nutritional outcome; arthroplasty/prosthesis/implant uses of the term 'Triathlon'; non-primary publications used only for context; and records without interpretable triathlon-specific data.

Supplementary identification and unavailable sources: Backward and forward citation tracking was used, but its source-specific yield was not separately logged. SPORTDiscus, Scopus, Web of Science, Embase, grey literature, conference abstracts, theses, and unpublished federation data were not systematically searched. This restricts claims of completeness

Table S1. Full study-level evidence inventory.

Report	Cohort flag	Population (sex; age; *n*)	Level/distance	Design/evidence type	Primary outcome/site	Principal contribution and main bias/limitation
Smith & Rutherford, 1993 [9]	—	Men; adult age NR; *n* = 8 triathletes	Athletes; distance NR	Cross-sectional comparison	DXA spine and total-body aBMD; testosterone	No lower group-level aBMD despite lower testosterone. Very small subgroup, cross-sectional design, and older DXA technology limit causal or contemporary clinical inference.
Morel et al. 2001 [10]	—	Men; adults; triathlon subgroup within *n* = 704 sportsmen	Amateur; distance NR	Mixed-sport cross-sectional	Site-specific DXA aBMD	Supports sport- and site-specific interpretation. Triathlon subgroup detail is limited and the analysis was not designed around triathlon-specific clinical risk.
Duncan et al. 2002 [11]	A†	Women; adolescents; *n* = 15 triathletes	Adolescent athletes; distance NR	Mixed-sport cross-sectional	DXA aBMD at multiple sites; muscle strength	Findings depended on site and loading modality. Small developmental subgroup; possible overlap with [12].
Duncan et al. 2002 [12]	A†	Women; 15–18 years; *n* = 10 triathletes	Adolescent athletes; distance NR	Mixed-sport cross-sectional	Mid-femur geometry / biomechanical properties	Geometry adds information beyond aBMD. Small subgroup and possible overlap with [11].
McClanahan et al. 2002 [13]	—	Men *n* = 9; women *n* = 12; adult age NR; total *n* = 21	Competitive; distance NR	24-week prospective season	Total-body and regional DXA aBMD	No adverse total-body or leg change; arm aBMD increased in men. Short follow-up and small sex strata do not establish long-term BSI safety.
Maïmoun et al. 2003 [14]	B‡	Men; young adults; *n* = 14 triathletes	Endurance athletes; distance NR	Cross-sectional comparison	DXA aBMD; testosterone	Reduced testosterone was not accompanied by lower group-level aBMD. Small sample and potential overlap with [16,17].
Nevill et al. 2004 [15]	C§	Men; adults; triathlon subgroup within *n* = 106 athletes	Multiple sports; distance NR	Mixed-sport cross-sectional	Site-specific DXA aBMD	Adjusted aBMD varied by sport and site. Triathlon subgroup detail is limited; dataset may overlap with [31].
Maïmoun et al. 2004 [16]	B‡	Men; adults; *n* = 14 triathletes within *n* = 38 athletes	Competitive; distance NR	Cross-sectional comparison	DXA aBMD and bone-turnover markers	Compatible with site-specific loading effects. Small subgroup and potential overlap with [14,17].

Maïmoun et al. 2004 [17]	B‡	Men; mean 19.3 years (18–20); n = 7	Competitive; distance NR	32-week longitudinal season	Lumbar-spine DXA aBMD; bone-turnover markers	Lumbar-spine aBMD increased 1.9% while a formation marker decreased. Very small sample, short follow-up, and potential cohort overlap.
Hoch et al. 2007 [18]	—	Women; 35 ± 6 years; n = 15	Club triathletes; distance NR	Cross-sectional	Triad characteristics; 3-day diet; total hip and L2–L4 aBMD	Energy and menstrual-history signals coexisted with normal group mean hip/spine aBMD. Small sample and short dietary window.
Wulff Helge et al. 2012 [19]	—	Women; adult age NR; n = 8 triathletes plus n = 7 controls	Elite; distance NR	Cross-sectional	DXA aBMD; sex hormones; isokinetic torque	aBMD related more clearly to muscle torque than one-time sex-hormone concentrations. Very small groups and one-time assays.
Newsham-West et al. 2014 [20]	—	Sex mixed/NR in summary; elite-development; n = 15	Elite-development; distance NR	Observational imaging	Regional tibial geometry; tibial stress reactions	Local cortical geometry associated with stress reactions. Observational association does not validate a screening threshold.
Ramos-Campo et al. 2015 [21]	—	Men; adult age NR; n = 18	Trained; distance NR	Controlled training study	DXA aBMD after simulated-altitude program	No clear short-term aBMD benefit. The intervention did not test LEA treatment; small sample and short duration.
Vescovi & VanHeest, 2016 [22]	—	Woman; elite junior; n = 1	Elite junior; distance NR	Longitudinal case study	Estimated EA; menses; hormones; lumbar-spine Z-score	Very low estimated EA coincided with endocrine suppression, menstrual dysfunction, and low lumbar Z-score. One case cannot estimate frequency or effect size.
Yi et al. 2016 [23]	—	Women; ≥18 years; n = 311	Self-selected triathletes; mixed distance	Online cross-sectional survey	Triad screening; no objective bone imaging	Twenty-four percent screened positive for at least one Triad arm. Self-selection, incomplete questionnaire response, and self-report limit diagnostic specificity.
Neidel et al. 2019 [24]	—	Sex mixed; adult age NR; n = 86	German elite; distance NR	Retrospective cross-sectional	Self-reported stress-fracture history and associated factors	Twenty reported ≥ 1 stress fracture. Recall, survivor selection, variable imaging confirmation, and uncertain temporality preclude incidence or causal inference.
Wu & Huang, 2019 [25]	—	Men; adult age NR; n = 9	Amateur; 226-km Ironman	Repeated measures around race	Osteocalcin and C-terminal telopeptide	Shows an acute post-race biochemical response. Sampling timing, hydration, feeding, and surrogate endpoints prevent long-term skeletal inference.
Gama et al. 2022 [26]	D¶	Women; adult age NR; n = 23 triathletes plus n = 17 controls	Long-distance	Cross-sectional	Estimated EA; DXA aBMD; distal radius/tibia HR-pQCT; fracture history	Favorable exercise-associated features appeared attenuated with low EA. Cross-sectional exposure, residual confounding, and self-reported injury history limit causality; possible link to [32].

Witkoś et al. 2023 [27]	—	Women; adult age NR; *n* = 30	Mixed level/distance NR	Cross-sectional questionnaire	LEAF-Q and menstrual-risk signals; no imaging	Identifies athletes warranting assessment but does not diagnose LEA, REDs, or low aBMD. Questionnaire and selection bias are possible.
Crunkhorn et al. 2024 [1]	—	Women and men; elite adult age NR; *n* = 50	Elite short-course; one Australian program	Four-year prospective surveillance	Clinician-recorded BSI incidence and time-loss burden	Twenty athletes (40.0%) had ≥ 1 BSI; burden 31.38 days/365 athlete-days (95% CI 24.42–38.34); female-to-male IRR 2.99 (95% CI 1.26–7.07). Strongest clinical design but narrow setting and no aligned nutrition exposure.
Hiromatsu & Goto, 2024 [28]	—	Men; elite adult age NR; *n* = 5	Elite; training camp	Case series / field physiology	Estimated EA and nocturnal interstitial glucose; no bone endpoint	Demonstrates male field measurement but cannot estimate skeletal risk or define an LEA threshold.
Cruz Correa Netto Soares et al. 2024 [29]	—	Women; adult age NR; *n* = 23 triathletes plus *n* = 19 inactive controls	Amateur; distance NR	Cross-sectional	Absolute and body-size-adjusted DXA aBMD	Interpretation changed with body-size adjustment. Research ratios are not validated clinical thresholds; cross-sectional design limits causality.
Langa et al. 2025 [30]	—	Women; adult age NR; *n* = 20	High-performing amateur; distance NR	Cross-sectional dietary assessment	Nutrient intake / REDs-related risk; no objective bone endpoint	Potential intake gaps were identified. Few recording days and reporting error limit inference about chronic EA or skeletal outcome.
Nevill et al. 2025 [31]	C§	Sex mixed; adult athletes across eight sports; triathlon subgroup NR	Multiple sports; distance NR	Cross-sectional allometric reanalysis	Body-mass-adjusted site-specific DXA aBMD	Shows site and size dependence. Triathlon subgroup detail and possible overlap with [15] limit independence.
Gama et al. 2026 [32]	D¶	Woman; professional adult age NR; *n* = 1	Professional; distance NR	Longitudinal case report	Triad recovery; menses/reproduction; bone outcomes	Reduced training and increased intake accompanied recovery. Temporal coherence is informative but cannot isolate the active component; possible cohort linkage to [26].

All 25 retained primary reports are listed. 'NR' indicates that the characteristic was not reported in the manuscript-level evidence inventory; no value was inferred. Cohort flags identify probable or possible overlap, not confirmed participant identity. Cohort flags: A†, potential Duncan overlap [11,12]; B‡, potential Maïmoun overlap [14,16,17]; C§, potential Nevill dataset overlap [15,31]; D¶, possible Gama cohort linkage [26,32]. Because recruitment dates and participant identifiers were incomplete, 25 reports are estimated to represent approximately 20–22 potentially independent cohort groups.

Table S2. SANRA author self-assessment.

SANRA item	Score (0–2)	Evidence in the revised manuscript
1. Justification of importance	2	Introduction defines the triathlon-specific clinical gap and why evidence domains require separation.
2. Concrete aims / questions	2	Introduction states four explicit review questions.
3. Description of literature search	2	Section 2 and Supplementary Methods S1 report database, dates, complete syntax, record count, purposive selection, exclusions, and limitations.
4. Referencing	2	Direct triathlon evidence is referenced separately from consensus and broader contextual literature; study-level inventory is supplied.
5. Scientific reasoning	2	Claims are weighted by directness, design, measurement validity, temporality, confounding, and cohort independence; causal limits are explicit.
6. Appropriate presentation of endpoint data	2	Precise study-specific endpoint data are reported where available; Tables 1–4 and Supplementary Table S1 distinguish outcomes and limitations.
Total	12/12	Author self-assessment of reporting quality; not a formal risk-of-bias judgment or guarantee of review completeness.

Supplementary Note. Interpretation of review scope

This critical narrative review does not claim exhaustive retrieval, duplicate screening, formal risk-of-bias grading, or pooled quantitative synthesis. The supplementary material is intended to make the authors' search and narrative weighting auditable while preserving the design stated in the manuscript.
